# Should monitoring guidelines and decision to treat for I⁠gM MGUS be different when caring for Jehovah’s Witness patients?

**DOI:** 10.46989/001c.158440

**Published:** 2026-04-23

**Authors:** Marshall Rosenberg, Alisha P. Maity, Karthik Shankar, Caroline Dunne, Arezoo Ghaneie

**Affiliations:** 1 Department of Internal Medicine, Lankenau Medical Center 100 E Lancaster Ave, Penn Wynne, PA 19096, USA; 2 Department of Hematology-Oncology, Lankenau Medical Center 100 E Lancaster Ave, Penn Wynne, PA 19096, USA

**Keywords:** MGUS, Waldenström’s Macroglobulinemia, hyperviscositysyndrome, Myeloma, Jehovah’s Witness

## Abstract

A practicing Jehovah’s Witness with diagnosed IgM monoclonal gammopathy of undetermined significance (MGUS) in 2014 was hospitalized within one year of her annual checkup. She had hyperviscosity syndrome with blurry vision and severe anemia but, due to religious beliefs, would not accept blood products. She was treated with therapeutic plasma exchange therapy with subjective improvement of her blurry vision, decrease in her serum viscosity, and improvement of her anemia. This bloodless medicine patient (BMP) had annual follow up per the Mayo Clinic Stratification System guidelines for her asymptomatic MGUS, as she fell into the intermediate-risk category given her age, laboratory values, and lack of symptoms. Due to the inability to provide proper rescue therapy with blood products in the event of anemia, we suggest adjusting the monitoring and surveillance interval for BMPs regardless of laboratory values. We suggest that BMPs undergo additional monitoring including iron and coagulation studies to preempt and prevent potential complications of symptomatic anemia, coagulopathy, and hyperviscosity syndrome. We recommend shortening the follow-up for these patients to every 3-6 months, as opposed to the standard follow-up that is recommended for IgM MGUS (typically ranging from 6-12 months). This would also drive earlier conversations about the risks and benefits of initiating earlier treatment in these patients, given their risks of complications if their MGUS progresses to malignancy.

## Introduction

IgM MGUS is a pre-malignancy that can progress to Waldenström Macroglobulinemia, AL Amyloidosis and, rarely, Multiple Myeloma.^1^ The Mayo Clinic’s stratification model predicts a 20-year risk of MGUS progression and utilizes serum M protein levels above 1.5 g/dL, non-IgG subtype, and abnormal free light chain ratio as risk factors. More numerous risk factors increase the likelihood of progression to disease from low, intermediate, and high risk, with 5%, 20% and 60% progression risk, respectively. The current standards for low risk MGUS follow up is every 2-3 years, and those with any additional risk factors should undergo 6-month monitoring at first and, if no abnormalities are seen on complete blood count, serum electrophoresis, and a basic metabolic count, can be moved to annual appointments.^1–3^ However, there is no current distinction in monitoring intervals in bloodless medicine patients, specifically patients who cannot receive first-line rescue therapy for anemia. For these reasons, this patient population is at increased risk of complications based on current guidelines.^4^

IgM MGUS also carries a higher risk of progression compared to other gammopathies, and increased risk of hyperviscosity syndrome at IgM protein levels above 0.6g/dL.^1,2^ These patients can exhibit bleeding, vision loss, heart failure, stroke, and coma.^5,6^ Additionally, patients with IgM MGUS that progress to Waldenström Macroglobulinemia or IgM Myeloma are at increased risk of anemia, due to the disease’s inherent bone marrow suppression and increase in hepcidin levels inhibiting gut absorption of enteric iron.^7^

## Case Report

A 74-year-old patient with bone marrow biopsy proven IgM lambda MGUS (original M protein level 0.22g/dL) and chronic lymphocytic leukemia in 2014 presented to the emergency room due to worsening blurry vision and an outpatient hemoglobin of 4.8g/dL. Six weeks prior, she had started to experience blurry vision in both eyes and was found to have papilledema on a fundoscopic exam. Her hemoglobin at this time was 11.6g/dL. She endorsed fatigued and intermittent shortness of breath, but denied lightheadedness, dizziness, hematemesis, melena, or hematochezia. In the emergency department, she was hypertensive to 173/75 mmHg and tachycardic to 107 beats per minute. Table 1 illustrates her initial laboratory values upon admission, demonstrating normocytic anemia, acute kidney injury, prolonged PT and PTT, and low iron and B-12 levels.

**Table 1.** Patient’s initial laboratory values obtained on admission

**Laboratory Test**	**Patient’s Value**	**Reference Range (Female)**
**Hgb**	4.8g/dL	12.0-16.0g/dL
**WBC**	15.3 x 10^9^/L	4.0-10.0x10^9^/L
**MCV**	94fL	80-100fL
**Creatinine**	0.13mg/dL	0.5-0.9mg/dL
**Iron level**	35 x10-7mcg/dL	80-180 x10-7mcg/dL
**TIBC**	237 x10-7 g/dL	270-460 x10-7g/dL
**Iron Saturation**	15%	15-45%
**Ferritin**	11µg/L	20-300µg/L
**Coombs test**	negative	negative
**Fecal Occult Blood Test**	positive	negative
**Total Bilirubin**	0.7mg/dL	0.3-1.2mg/dL
**Lactate dehydrogenase**	76IU/L	<220IU/L
**Haptoglobin**	111.0U/L	41.0-203.0U/L
**Absolute Reticulocyte Count**	10.0x10^9^/L	25-100x10^9^/L
**Reticulocyte Index**	0.38%	0.60-2.80%
**Vitamin B-12**	59.8pg/mL	>350pg/mL
**Folate**	15ng/mL	3-14.7ng/mL
**TSH**	2.63mIU/L	0.34-5.60mIU/L
**PT**	22.4seconds	12.2-14.5seconds
**PTT**	53seconds	23-35seconds
**INR**	1.7	0.8-1.1

Hgb=hemoglobin, WBC=white blood cells, MCV=mean corpuscular volume, INR=international normalized ratio, TIBC=total iron binding capacity, LDH=lactate dehydrogenase, TSH=thyroid stimulating hormone, PT=prothrombin time, PTT=partial thromboplastin time.

**Figure 1.**
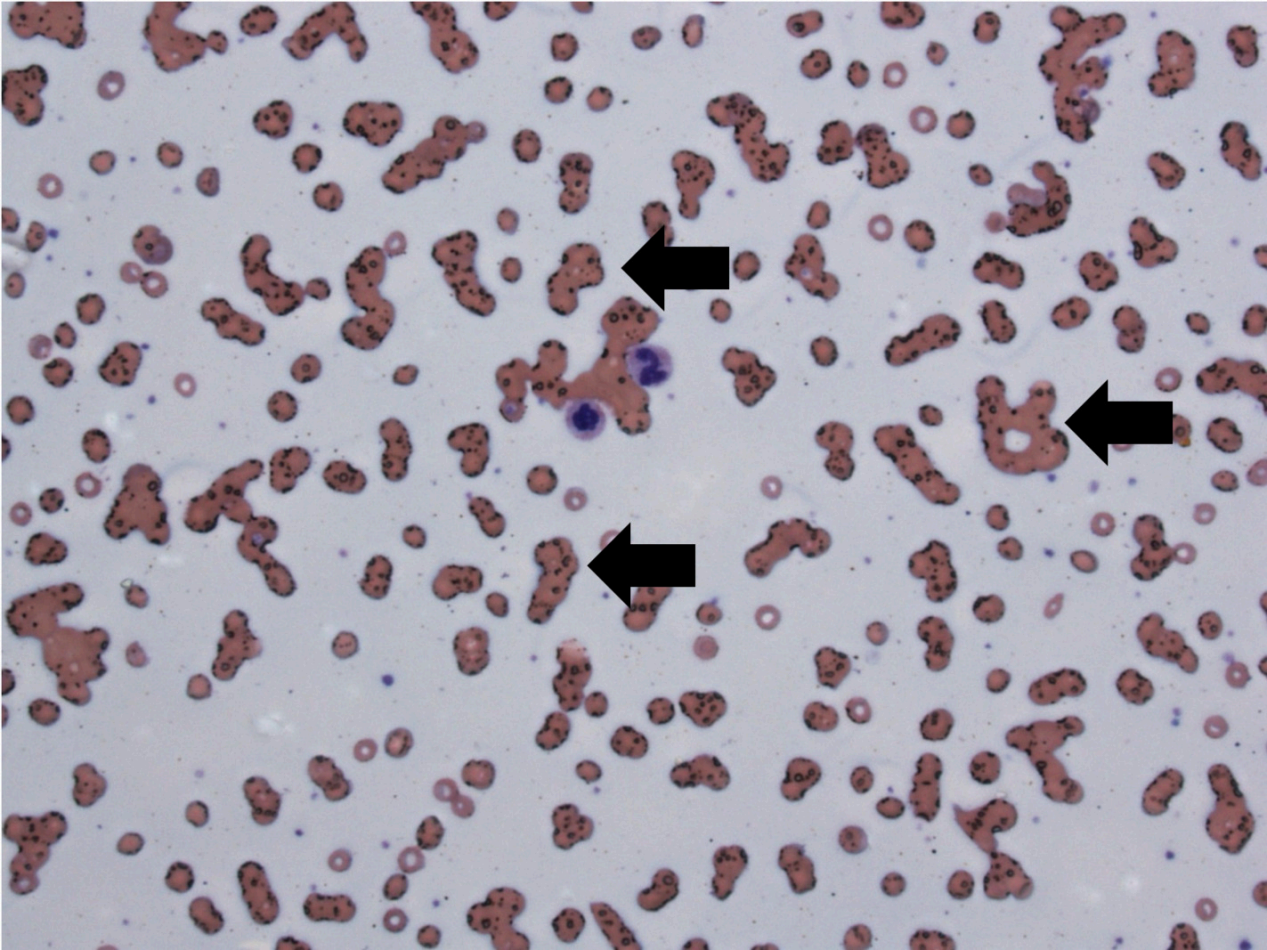
Rouleaux formations seen on 10x blood smear (black arrows)

Admitted out of concern for a gastrointestinal bleed, she was unable to safely undergo a colonoscopy due to her severe anemia. As a Jehovah’s Witness, she would not accept packed red blood cell transfusions. Therefore, to help correct her anemia and vitamin deficiencies, she received iron, erythropoietin, vitamin B-12, and folate. Figure 1 depicts her peripheral smear from her second day of admission that showed evidence of cold rouleaux formations.

Due to these findings on her blood smear, serum protein electrophoresis and immunofixation were ordered (Table 2).

**Table 2.** Results of serum protein electrophoresis and immunofixation

Laboratory Test	Patient’s Value in 2014	Patient’s Value During Hospitalization	Reference Range
M protein	0.22g/dL	5.89g/dL	0.37-2.8g/dL
Kappa Free Light Chain	8.8mg/L	115mg/L	3.3–19.4mg/L
Lambda Light Chain Value	7.3mg/L	5.3mg/L	5.71–26.3mg/L
Kappa/Lambda Ratio	1.2	21	0.26-1.65
IgM	301mg/dL	8698mg/dL	37–286mg/dL
IgG	1124mg/dL	211mg/dL	767–1,590mg/dL
IgA	122mg/dL	28mg/dL	61–356mg/dL
β₂-microglobulin	1.64 mg/L	4.56mg/L	0.98-2.52mg/L

IgM=immunoglobulin M. IgG=immunoglobin G, IgA=immunoglobulin A.

**Figure 2.**
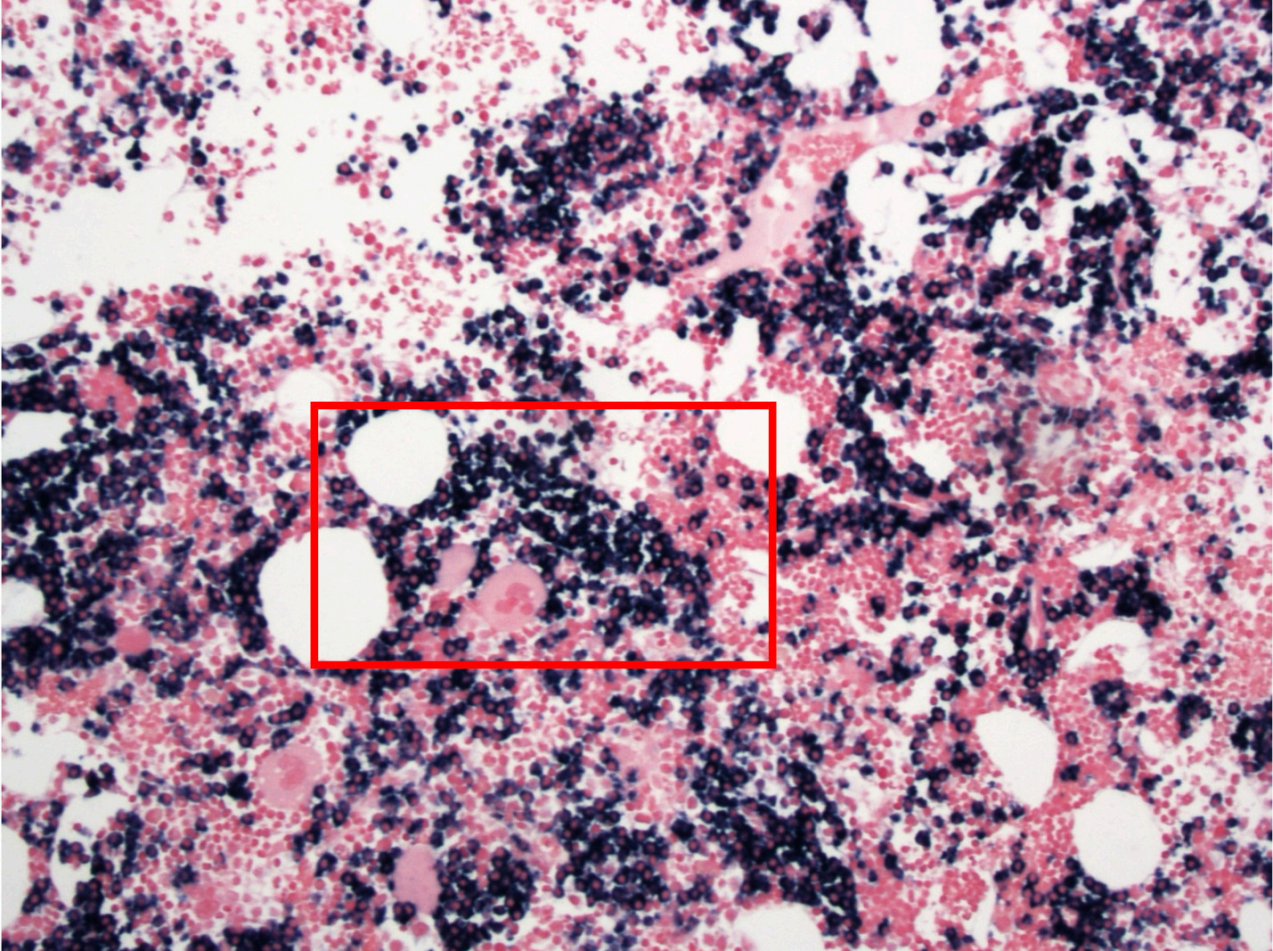
Bone marrow aspirate displaying Kappa restricted stained black cells (red box)

While no episodes of melena or hematochezia occurred during her hospitalization, the patient unfortunately experienced worsening vision on day 7. Computed tomography of the head and magnetic resonance imaging of the brain performed out of concern for cerebral vascular accident did not show evidence of acute intracranial abnormalities. Fundoscopy revealed scattered dot blot hemorrhages, subretinal hemorrhages, and cotton wool spots scattered throughout the fundi of both eyes. Out of concern for hyperviscosity syndrome, serum viscosity was obtained and was greater than 7.9cp (reference range:1.4-1.8cp). These findings confirmed hyperviscosity syndrome and, thus, she was treated with plasma exchange therapy with albumin for volume replacement. The patient underwent two sessions over the next two days and reported subjective improvement of her blurry vision. On day 9 of hospitalization, her serum viscosity decreased to 2.1cp, her hemoglobin rose to 6.9g/dL, PT decreased to 16.5 seconds, and PTT decreased to 37 seconds. During this hospitalization there were no episodes of melena, hematemesis or hematochezia, decreasing the likelihood of an ongoing gastrointestinal bleed. Bone marrow biopsy performed during the hospitalization revealed marrow focally replaced by sheets of neoplastic lymphocytes and plasma cells. Figure 2 shows the patient’s bone marrow aspirate with kappa-restricted neoplastic plasma cells that comprised approximately 30% of marrow cellularity.

The plasma cells were also positive for Mum 1 and cyclin D1, weakly positive for CD20 and PAX5, but predominantly negative for CD56. CD3 appears on scattered small mature T cells. There was also another population of neoplastic B cells positive for CD20 and PAX5 (strongly), CD5 and LEF1, but negative for cyclin D1 and SOX11. There were no increased blasts. Fluorescent in situ hybridization revealed an immunoglobulin heavy chain gene rearrangement at 8% with CCND1 (BCL1/IGH t(11;14)). Mutational analysis was negative for MYD88 and CXCR4, supporting the diagnosis of IgM kappa myeloma or IgM myeloma/Waldenström Macroglobulinemia overlap syndrome. On day 16, the patient was discharged with plans to begin systemic treatment with cyclophosphamide, bortezomib, dexamethasone, and daratumumab.

## Discussion

For patients with IgM MGUS, the Mayo Clinic Stratification System utilizes a risk factor scoring system to predict progression of disease in 20 years.^8,9^ This patient fell into the low-intermediate category, with only IgM gammopathy as the risk factor that would recommend annual checkups, after an initial biopsy with FISH analysis was completed that was normal. At the time of increased IgM detection, her outpatient oncologist utilized the International Prognostic Staging System (IPSS) to predict the utility of treating individuals with Waldenström macroglobulinemia, as it was presumed she would progress to this condition due to its common occurrence with elevated IgM. Poor prognostic factors include a patient over the age of 65, a hemoglobin count less than 11.5g/dL, platelet count less than 100X10^9^/L, a β₂-microglobulin concentration greater than 3mg/L, or M-protein concentration greater than 7.0g/dL.^8^ The number of prognostic factors stratify patients into low-risk, intermediate and high-risk categories. If a patient less than 65 years old presents with 0-1 bad prognostic factors, there is a 5-year 87% survival rate, and this rate decreases to 68% in intermediate groups, and 36% in high-risk groups. At age 74, she had 1/6 bad prognostic values and, based on these factors, she and her oncologist elected watchful waiting before treatment.

While the Mayo clinic risk stratification and IPSS systems can be used in typical patient populations, Jehovah’s Witnesses have religious beliefs derived from scripture, and often refuse the transfusion of all major blood products.^10^ For patient populations who do not accept blood products, their risk of complications if anemia, thrombocytopenia, amyloidosis, or cryoglobulinemia develop presents a unique challenge.^4,11^ This patient was hospitalized 11 months after her annual oncology appointment. Based on her specific predicament, when stratifying this sort of patient to determine the risk of progression to help inform decision to initiate treatment, we would recommend elevating the risk score to increase the frequency of outpatient follow-up visits. When utilizing the Mayo Risk Score for IgM MGUS, all follow up appointments after the initial 6-month interval should indefinitely remain at 6 months, versus extending to annual visits. This would entail moving annual follow up for low-risk patients to the 6-month intervals seen in intermediate risk patients, and change 6-month follow up intervals to 3 months used for high-risk patients.

These special patient populations should also have their iron levels examined during these checkups, to monitor the need for intravenous iron supplementation. Bone marrow suppression causes anemia and, while the exact mechanisms remain unclear, increased hepcidin levels seen in this disease also inhibit iron transfer from enterocytes and macrophages, leading to iron deficiency anemia refractory to oral iron.^7^

Close attention to the presence of constitutional symptoms, bleeding, lightheadedness, confusion, headaches, and vision symptoms are vital to review in any patient with an IgM gammopathy, given the risk of hyper-viscosity syndrome. These office visits should include complete blood count with iron studies to monitor for anemia and iron deficiency, serum protein electrophoresis with immunofixation to monitor IgM paraprotein levels, and coagulation studies that act as early warning signs pointing to progression to disease. It is worthwhile to utilize these tests alongside more frequent office visits as they will assist in earlier discussions between patients and their oncologist about the risks and benefits of early treatment initiation.

### Authors’ Contribution per CRediT

A.P.M. and A.G and M.R conceptualized the case report, M.R and A.P.M wrote the draft manuscript, K.S, C.D and A.G edited the manuscript, M.R and A.P.M revised the manuscript. All authors approved the final manuscript.

### Competition of Interest – COPE

No competing interests were disclosed.

### Informed Consent Statement

All authors and institutions have confirmed this manuscript for publication. Written consent was received from the patient to publish this work.

## Data Availability

All are available upon reasonable request.
